# Is There an Ideal Payment Model That Enables Integrated Care: Searching for the Impossible?

**DOI:** 10.5334/ijic.9029

**Published:** 2024-11-22

**Authors:** Volker Amelung, Jason Cheah

**Affiliations:** 1 Hannover Medical School (MHH) for management in health care, Hannover, Germany; 2 Lee Kong Chian School of Medicine Nanyang Technological University, Singapore

**Keywords:** payment models, capitation, value-based

[1] As the science (and art) of integrated care evolves and expands [3], the one thing that is as yet unresolved is how best to finance integrated care in a sustainable way. Payment models encompass a wide array of archetypes and target groups. There are payment models between providers, from patients to providers, from patients to payors (eg insurers and other intermediaries), and from payors (eg government, insurers and other intermediaries) to providers [4]. This editorial focuses mainly on the last, ie payment models used by payors as a means to fund providers of health and social care services to integrate care.

In few other areas of health system research is it so obvious how differently people can think, even though they believe they are talking about the same thing. Representatives of Beveridge-style state health systems tend to understand reimbursement systems quite differently from those of social insurance systems or market models. When it comes to state health systems, the focus is on budgeting and the associated governance structures, while for the others it is on pricing models.

Interestingly, the two lines of reasoning converge somewhat when it comes to ‘global budgets and capitation models’ in integrated care. Although the content is largely identical, the logic is different: for one it is a business case, for the other it is a system logic or governance structure. Misunderstandings are inevitable here, or rather, people are talking about the same thing with completely different understandings. This is particularly interesting when you look at the large number of pilot projects on ‘global budgets’ such as in Scotland, England, the Netherlands, Finland or Spain. It is not crucial whether one or the other perspective is the right one, but we must above all be aware of the different perspectives. The following remarks focus primarily on compensation systems and thus tend to have a microeconomic perspective.

Remuneration systems for integrated care face various fundamental challenges. In medicine, we are accustomed to reimbursing for discrete or episodic services, such as a mammography, but in integrated care, the nature of services is much more complex. We would like to emphasize five points:

1. Integrated care often requires very long-term investments, i.e. the effects and outcomes only occur after a considerable period of time. Integrated care is based on the paradigm that better care usually also means more cost-effective care. A well-cared-for diabetic patient, for example, requires significantly fewer services (e.g. avoidable amputations), which means that a payment system for integrated care must always include a quality component that positively rewards the avoidance of services in a longer term perspective.
2. Integrated care necessitates coordination and communication between different stakeholders and sectors – whether in person or via digital platforms. Traditionally, however, many healthcare systems do not directly account for such activities. In integrated care, management is an essential task that should also be recognized and remunerated accordingly.
3. Integrated care is based on a team approach that leads to a new distribution of roles and corresponding shifts in the respective budgets. The delegation and substitution of tasks should not be at the expense of individual professional groups; rather, the goals of all stakeholders must be taken into account.
4. Integrated care is usually a complex intervention, and it is extremely difficult to determine who is responsible for which success. Therefore, we need a payment system, which does not focus on ad hoc activities, but long term effects with often difficult to judge impact of the different players.
5. Probably the biggest challenge in the payment models for integrated care is the mismatch between costs and savings. Particularly in social insurance systems, the costs often arise in the health insurance arena, while the benefits accrue to, for example, in pension funds or even employers. Investments in integrated care can then become irrational for the payor system, and compensation mechanisms must be developed in a much more holistic approach.

There are numerous payment models that are currently being used/implemented in different health and care systems [5]. Yet, there doesn’t seem to be an ideal model or method that satisfactorily factors all the five key considerations as outlined above. Payment models, in practice, are always considered as a second best solution, either focusing too much on cost control or quality assurance. Therefore, it is always a question of optimization, which is in practice is a real challenge. No system has claimed so far to have found a “perfect” balance and solution. Interestingly, some of these failed approaches seem to be coming back just 15 years later, and are being touted as “new” solutions.

In the US, the dominance of the fee-for-service payment model and more recently, again capitation payment models of varying types and combinations have evolved to a highly complex system. In Europe and other more social insurance based or socialist systems, payment models are generally constructed between practising physicians, providers (eg hospitals) and insurers. In other systems (eg in Singapore), a complex set of mixed models have evolved, which tries to strike a balance between efficiency (output based payment models) and quality (value-based care incentives). Regardless of the model, there is clearly no one payment model that seems to be able to satisfy the *ideal* financing approach of:

- Equity
- Efficiency
- Effectiveness
- Enabling best patient outcomes

In trying to understand the wide realm of payment models on a micro economic level, there seems to be four commonly used archetypes:

(a) Fee-for-service (FFS).
(b) Bundled payments.
(c) Per-individual (membership) payments.
(d) Incentive payments.
(e) Blended models (any combinations of (a) to (c), with or without (d)).

But most health and care systems, the key principles in designing these models include:

(a) Value-Based Payments, ie paying for value and outcomes, instead of workload or volume driven payments. Based on the core idea of value based health care [2], not volume but outcomes should be reimbursed. This idea is highly attractive for all actors in the health care system – it fits to the values of providers, the goals of purchasers and is also in the interest of patients. At first glance, it looks like a perfect solution. In practice, however, it is clear that it places the greatest demands on the measurement methodology and must be risk-adjusted.
(b) Incentivise providers to “do the right things”. This could include encouraging providers to adhere to national care protocols/guidelines, providing appropriate care to patients at the most cost-effective settings, enable integrated or coordinated care, and optimise capacities and capabilities.
(c) Risk Adjusted Payments. This is most advanced in the US. The general principle being to allow providers to plan and be appropriately resourced to serve the casemix of patients, but not be overly complicated for implementation, as not all providers have the same level of capabilities.
(d) Facilitating Sector Development. It is thought that some payment models should facilitate the establishment of strong partnerships and sharing or transfer of capabilities to enable localised community sector development.
(e) Shared Savings and Risks. This is a relatively new development, mainly in the US. The idea here is that when there is alignment of goals and interests between payors and providers (and ideally, even patients), whatever financial incentives and surpluses gained from integrated care programmes should be shared between the various parties concerned. Thus far, these have been implemented as discrete pilot programmes.

Most payment models are based on socio-political constructs and philosophies. As such, it would be naïve to try to impose specific payment models without due consideration for the five principles stated above.

The traditional and “tried and tested” fee-for-service (FFS) and output based payment models have been opined and proven to be effective in driving throughput and higher efficiencies (eg DRG-based payment models), but these do not encourage care to be joined up/integrated across settings, resulting in sub-system optimisation but overall system fragmentation.

So with all that has been implemented and researched thus far, and with the myriad of payment models in existence today, what can we learn and what can we convincingly say “works” and what “doesn’t work”? In the realm of integrated care, various payment models have been promulgated including global budgets, shared/bundled payment models, and capitation. While conceptually sound and rational, the evidence is still uncertain on the longer term impact of these on care integration and care outcomes, mainly because of the multitude of factors which affect/impact on clinical, functional and social outcomes. Capitation, as a means of paying providers to “go upstream”, and focus increasingly on prevention (and consequently screening and early detection of chronic illnesses and cancers) does seem to be increasingly popular and conceptually sound. However, the jury is still out in terms of whether or not capitation based payment models indeed do result in better population and patient based outcomes. Most of the published studies thus far have mainly focused on processes of care related to some form of capitation-based financing. However, the experiences from the USA should be taken into account. After an absolute hype phase during the managed care period at the end of the last century, capitation contracts have declined significantly. In particular, the exploitation of market power is a risk factor for capitation, in that remuneration has been systematically reduced. Thus, capitation only works in healthcare systems in which the various players treat each other fairly.

Payment models and Value Based Health Care (VBHC) are often intricately linked and need to be contextualised to each health/social care system. In the literature and conferences, there is an increasing interest in Value Based Payment or Financing Models. These may take many forms but typically link some form of payment model with specific reportable outcomes (eg patient-based, clinical or functional). Thus far, it has been difficult to translate and spread or translate the same model from one country to another unless the organisation and financing systems are almost the same.

Much has been written and said on the need to design incentive structures into payment models. It is clear from most policy makers and payors that incentives are important. Is there a “magic number”? Based on the authors’ discussions, it seems to be somewhere between at least 10–20% of the physician/clinic/hospital/provider earnings in order for VBHC and payment models to make any impact on provider behaviours and practices. Designing incentive models is also gaining increasing attention as health and care systems and payors grapple with ever increasing pressures and constraints on resources and manpower.

With the increasing global interest in integrated care, it is imperative that each health and social care system should design, devise and implement the most cost-effective and sustainable payment model(s) that would be best suited in it’s socio-political and economic context. Rationally and prudently selecting from the array of payment models available today is critical if integrated care is to be implemented in a financially sustainable manner. There is clearly no “magic bullet” nor one best model. A great deal depends on the balance between the four key principles and also the political power and influence of providers and payors, who are often at odds with each other. With that, there is much room for creativity and transformation in payment models, and this is an area where academics, policy makers, payors and providers could come together to make a real difference. The real goal is an integrated care system that is sustainable.

We need robust studies to be done on the *impact* of payment models on improving clinical, functional and other health and social care related outcomes. These studies need to take into account the context of the health and care system in which the study is carried out.
